# Comparative proteomics dataset of skimmed milk samples from Holstein and Jersey dairy cattle

**DOI:** 10.1016/j.dib.2016.01.038

**Published:** 2016-01-30

**Authors:** Rinske Tacoma, Julia Fields, David B. Ebenstein, Ying-Wai Lam, Sabrina L. Greenwood

**Affiliations:** a The University of Vermont, Department of Animal & Veterinary Sciences, 570 Main Street, Burlington, VT 05405, United States; b The University of Vermont, Vermont Genetics Network Proteomics Facility, 109 Carrigan Drive, Burlington, VT 05405, United States

**Keywords:** Bovine, Proteome, Milk

## Abstract

Milk samples were collected from Holstein and Jersey breeds of dairy cattle maintained under the same management practices and environmental conditions over a seven-day period. Milk samples were collected twice daily from six cows of each breed as previously described (Tacoma et al., 2016) [1]. Samples were composited within individual cow over the experimental period and skimmed to remove the fat layer. Skimmed milk samples were fractionated using CaCl_2_ precipitation, ultracentrifugation and ProteoMiner treatment to remove the high abundance milk proteins. Separation of the low abundance proteins was achieved using SDS-PAGE. Differential protein abundances were analyzed by mass spectrometry-based proteomic approaches followed by statistical analyses of the peptide count data. The complete list of low-abundance proteins identified in both breeds is provided in the dataset as well as the total number of distinct sequenced peptides and gene ontology functions for each protein. The relative abundance of a select few proteins is depicted using the SIEVE software.

## Specifications Table

**Table:** 

Subject area	*Biology*
More specific subject area	*Milk proteomics*
Type of data	*Table, figure*
How data was acquired	*Acquired by Linear ion trap* (*LTQ*)-*Orbitrap Discovery mass spectrometer coupled to a Surveyor MS Pump Plus* (*Thermo Fisher Scientific, Waltham, MA, USA*)*. Raw data searched using the SEQUEST search engine on Proteome Discoverer* 1.4 (*Thermo Fisher Scientific, Waltham, MA, USA*) *against a curated Bovine Uniprot* (-*Bos taurus database* (24,206 *entries*) *downloaded July* 9, 2014-)
Data format	*Analyzed*
Experimental factors	*Milk samples were collected from Holstein and Jersey dairy cattle, frozen in a dry-ice ethanol bath and stored at* −80 °C until they were thawed and pooled within cow according to yield. A protease inhibitor was added to each sample before the fat layer was skimmed and removed.
Experimental features	*Skimmed milk samples were fractionated using* CaCl_2_*for casein precipitation, ultracentrifugation, and ProteoMiner treatment. Proteins were separated on SDS-PAGE followed by in-gel tryptic digestion. Protein abundances were analyzed using nano LC–MS/MS. A subset of differentially expressed proteins were analyzed using SIEVE software, which plots the ion elution profile on the chromatographic time scale* (*extracted ion chromatograms*) *of the identified target peptides.*
Data source location	*Paul R. Miller Research and Educational Center, University of Vermont, Burlington, VT and The Vermont Genetics Network Proteomics Facility, University of Vermont, Burlington, VT*
Data accessibility	*Analyzed datasets are directly provided with this article*

## Value of the data

• Compares the diversity of the bovine milk proteome from two prominent North American dairy breeds maintained under the same diet, environment and management conditions in order to better assess true breed differences.
• Allows for more direct comparison between research being performed using different breeds of dairy cattle, and can allow for general extrapolation and application of results seen in one breed to another.
• Expands the bovine milk proteome and provides a platform for future research investigating milk proteomics.

## 1 Experimental design, materials and methods

### 1.1 Sample collection

Milk samples were collected from six Jersey cows (80±49 days in milk (DIM)) and six Holstein cows (75±21 DIM) maintained under the same management practices and environmental conditions over a period of seven days as previously described [1]. Cows were milked twice daily at 0400 and 1600 h. Milk yield was recorded at each milking and milk samples were collected during the morning and afternoon milking throughout the 7-day experiment. Milk subsamples collected for low abundance protein analysis were immediately frozen in a dry-ice ethanol bath after collection and stored at −80 °C until further analysis.

### 1.2 Enrichment of the low abundance proteins

Before analysis, milk samples were thawed overnight at 4 °C. To obtain a representative sample for each animal, aliquots were composited within animal across the week according to milk yield at each milking. A mammalian protease inhibitor cocktail (Protease Inhibitor Cocktail, Sigma, Milwaukee, WI, USA) was added at 0.24 ml inhibitor per gram of protein in the milk to a 50 ml aliquot sample which was then centrifuged at 4000×*g* for 15 min at 4 °C. The cream layer was removed and skimmed samples were depleted of casein using a previously described method [2]. Briefly, CaCl_2_ (60 mM) was mixed into the skimmed sample and the pH was adjusted to 4.3 using 30% acetic acid (Fisher Scientific, Fair Lawn, NJ, USA). Samples were then centrifuged at 189,000×*g* at 4 °C for 70 min and the supernatant was collected and stored at −80 °C [2], [3]. Samples were lyophilized and reconstituted to 500 mg whey powder in 1 ml PBS. The samples were analyzed for their protein content using the bicinchoninic acid assay kit (Pierce, Rockford, IL, USA), with bovine serum albumin as the standard. The low-abundance minor proteins were enriched by the ProteoMiner Kit (BioRad Laboratories, Hercules, CA, USA) and 32 mg of whey protein was added to 100 μl of ProteoMiner beads. The whey samples were gently shaken with individual ProteoMiner columns for 2 h at room temperature and columns were washed thoroughly using HPLC grade water to remove excess proteins [4], [5]. The low abundance proteins were eluted off the beads by addition of 20 μl 4× Laemmli sample buffer (8% SDS, 40% glycerol, 250 mM Tris, pH 6.8, 400 mM DTT with trace amount of bromophenol blue). The mixture of the protein solution with the beads was heated at 95 °C for 10 min and the supernatant was subjected to SDS-PAGE on a precast 8–16% polyacrylamide gel to separate proteins (BioRad, Hercules, CA, USA). Tris-glycine (pH 8.3) was used as the running buffer containing 0.1% SDS. Electrophoresis was performed for approximately 35 min at 200 V until the protein bands reached the bottom of the gel. Staining was performed using Coomassie Brilliant Blue (BioRad, Hercules, CA, USA) overnight. To visualize the protein bands, destaining with a solution of 10% acetic acid, 40% methanol, and water was performed until the background was clear. The SDS-PAGE stained gels were scanned with a Gel Doc XR+ system (BioRad, Hercules, CA, USA).

### 1.3 In-gel digestion

Gel lanes were cut into 15 slices along the migration path and cut into small 1mm cubes. The gel pieces were destained with 50 mM ammonium bicarbonate in 50% acetonitrile. Protein samples were reduced by 10 mM DTT at 55 °C for 1 h and alkylated with 55 mM iodoacetamide in the dark at room temperature for 45 min. The gel pieces were then washed/rehydrated and dehydrated twice alternately with 100 mM ammonium bicarbonate and 100% acetonitrile. The gel pieces were dried in a SpeedVac (Scientific Support, Hayward, CA, USA) and trypsin digestion was carried out for 18 h at 37 °C with 7 ng μl^−1^ of trypsin. The tryptic peptides were acidified with 50 μl of 5% formic acid to stop the reaction, extracted, and dried in a SpeedVac (Scientific Support, Hayward, CA, USA). The dried peptide samples were re-suspended in 10 μl of a solution of 2.5% acetonitrile and 2.5% formic acid in water for analysis by LC–MS.

### 1.4 Protein identification by nano-scale LC/MS

LC–MS based protein identification was performed on a linear ion trap (LTQ)-Orbitrap Discovery mass spectrometer coupled to a Surveyor MS Pump Plus (Thermo Fisher Scientific, Waltham, MA, USA). Samples (5 μl) were loaded onto a 100 μm×120 mm capillary column packed with MAGIC C18 (5 μm particle size, 20 nm pore size, Michrom Bioresources, CA, USA) at a flow rate of 500 nl min^−1^. Peptides were separated using a gradient of 5–35% acetonitrile/0.1% formic acid over 98 min followed by 35–100% acetonitrile/0.1% formic acid in 1 min and then 100% acetonitrile for 10 min, followed by an immediate return to 2.5% CH3CN/0.1% FA and a hold at 2.5% CH3CN/0.1% FA. Peptides were introduced into the linear ion trap via a nanospray ionization source and a laser pulled ~3 μm orifice with a spray voltage of 1.8 kV. Mass spectrometry data was acquired in a data-dependent “Top 10” acquisition mode with lock mass function activated (*m*/*z* 371.1012216635), in which a Orbitrap survey scan from *m*/*z* 360–1600 at 30,000 (FWHM) resolution was paralleled by 10 collision-induced dissociation (CID) MS/MS scans of the most abundant ions in the LTQ. MS/MS scans were acquired with the following parameters: isolation width: 2*m*/*z*, normalized collision energy: 35%, Activation Q: 0.250 and activation time=30 ms. Review mode for FTMS master scans was enabled. Dynamic exclusion was enabled (repeat count: 2; repeat duration: 30 s; exclusion list size: 180; exclusion duration: 60 s). The minimum threshold was 500. Singly charged ions were excluded for MS/MS. Product ion spectra were searched using the SEQUEST search engine on Proteome Discoverer 1.4 (Thermo Fisher Scientific, Waltham, MA, USA) against a curated Bovine Uniprot (-Bos taurus database (24,206 entries) downloaded July 9, 2014-) with sequences in forward and reverse orientations. The 15 raw files from each Holstein (6 samples) and the 15 raw files from each Jersey (6 samples) cows were searched as one contiguous input file and a single result file was generated for each (12 results files in total). Search parameters were as follows: full trypsin enzymatic activity, two missed cleavages, and peptides between the MW of 350–5000; mass tolerance at 20 ppm for precursor ions and 0.8 Da for fragment ions, dynamic modifications on methionine (+15.9949 Da: oxidation) (4 maximum dynamic modifications allowed per peptide); and static modification on cysteine (+57.0215 Da: carbamidomethylation). The result files were then further analyzed by Scaffold 4.3 (Proteome Software, Portland, OR, USA) to compare the unique peptide counts and to identify gene ontology functions of the identified proteins. Cross-correlation (Xcorr) significance filters were applied to limit the false positive rates to less than 1% in both data sets. The Xcorr values were as follows: (+1): 1.5, (+2): 2.2, (+3): 2.8, (+4): 3.5. Other filters applied were a minimum peptide cutoff of 2 as well as DeltaCN >0.1. Ultimately, the confidence parameters resulted in less than 1% false discovery.
