# Supplementary material for: Comparative proteomics dataset of skimmed milk samples from Holstein and Jersey dairy cattle
Source: Data Brief. 2016 Jan 30;6:843–6. doi: 10.1016/j.dib.2016.01.038 (PMC4749939; doi:10.1016/j.dib.2016.01.038)
Supplement: Supplementary file 1 — Supplementary material [file mmc1.zip › DiB_Data_Zip_File.zip!/DiB + Suplemental Data Zip File/Supplemental Figure 1 caption line #.docx]

Supplemental Fig. 1: SIEVE analysis of a select few proteins existing at significantly different abundance (A,B,C) and at abundace with insignificant difference (D,E,F) between breeds.
