# Supplementary material for: Comparative proteomics dataset of skimmed milk samples from Holstein and Jersey dairy cattle
Source: Data Brief. 2016 Jan 30;6:843–6. doi: 10.1016/j.dib.2016.01.038 (PMC4749939; doi:10.1016/j.dib.2016.01.038)
Supplement: Supplementary file 1 — Supplementary material [file mmc1.zip › DiB_Data_Zip_File.zip!/DiB + Suplemental Data Zip File/Supplemental Table 1 caption line #.docx]

Supplemental Table 1: Identification of gene ontology functions of the full protein profile between different breeds
